# 3D Printed Porous Nanocellulose-Based Scaffolds As Carriers for Immobilization of Glycosyltransferases

**DOI:** 10.1021/acsabm.2c00763

**Published:** 2022-12-05

**Authors:** Florian Lackner, Hui Liu, Andreja Dobaj Štiglic, Matej Bračič, Rupert Kargl, Bernd Nidetzky, Tamilselvan Mohan, Karin Stana Kleinschek

**Affiliations:** ‡ Institute for Chemistry and Technology of Biobased System (IBioSys), Graz University of Technology, Stremayrgasse 9, Graz8010, Austria; ⊥ Institute of Biotechnology and Biochemical Engineering, NAWI, Graz University of Technology, Petersgasse 12, Graz8010, Austria; || University of Maribor, Faculty of Mechanical Engineering, Laboratory for Characterization and Processing of Polymers, Smetanova Ulica 17, Maribor2000, Slovenia; # University of Maribor, Institute of Automation, Faculty of Electrical Engineering and Computer Science, Koroška cesta 46, Maribor2000, Slovenia; □ Austrian Centre of Industrial Biotechnology (ACIB), Graz8010, Austria

**Keywords:** nanofibrillated cellulose, carboxymethyl cellulose, citric acid, cross-linking, direct-ink-writing 3D printing, enzyme immobilization, glycosyltransferase, nothofagin

## Abstract

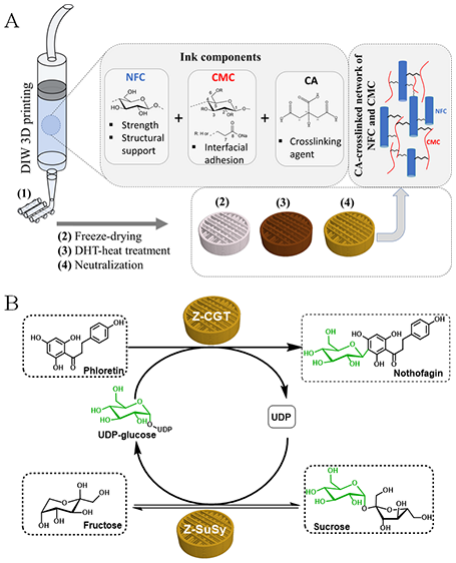

Biocatalysis is increasingly becoming an alternative method for the synthesis of industrially relevant complex molecules. This can be realized by using enzyme immobilized polysaccharide-based 3D scaffolds as compatible carriers, with defined properties. Especially, immobilization of either single or multiple enzymes on a 3D printed polysaccharide scaffold, exhibiting well-organized interconnected porous structure and morphology, is a versatile approach to access the performance of industrially important enzymes. Here, we demonstrated the use of nanocellulose-based 3D porous scaffolds for the immobilization of glycosyltransferases, responsible for glycosylation in natural biosynthesis. The scaffolds were produced using an ink containing nanofibrillated cellulose (NFC), carboxymethyl cellulose (CMC), and citric acid. Direct-ink-writing 3D printing followed by freeze-drying and dehydrothermal treatment at elevated temperature resulted in chemically cross-linked scaffolds, featuring tunable negative charges (2.2–5.0 mmol/g), pore sizes (10–800 μm), fluid uptake capacity, and exceptional dimensional and mechanical stability in the wet state. The negatively charged scaffolds were applied to immobilize two sugar nucleotide-dependent glycosyltransferases (C-glycosyltransferase, Z_basic2_-CGT; sucrose synthase, Z_basic2_-SuSy), each harboring a cationic binding module (Z_basic2_) to promote charge-based enzyme adsorption. Both enzymes were immobilized at ∼30 mg of protein/g of dry carrier (∼20% yield), independent of the scaffold used. Their specific activities were 0.50 U/mg (Z_basic2_-CGT) and 0.19 U/mg (Z_basic2_-SuSy), corresponding to an efficacy of 37 and 18%, respectively, compared to the soluble enzymes. The glycosyltransferases were coimmobilized and shown to be active in a cascade reaction to give the natural C-glycoside nothofagin from phloretin (1.0 mM; ∼95% conversion). All enzyme bound scaffolds showed reusability of a maximum of 5 consecutive reactions. These results suggest that the 3D printed and cross-linked NFC/CMC-based scaffolds could present a class of solid carriers for enzyme (co)-immobilization, with promising applications in glycosyltransferase-catalyzed synthesis and other fields of biocatalysis.

## 1 Introduction

Enzyme biocatalysis plays a key role in various applications, including pharmaceuticals, food, biomedicine, biochemistry, etc.^1−3^ For such applications, the enzymes immobilized on a suitable carrier (e.g., scaffold) are preferred over free enzymes (in solution), because they have the advantage of improving operational stability, cost efficiency, product separation, and enzyme reusability.^4−7^ Both physical (enzyme entrapment in the matrix) or covalent (intermolecular cross-linking between enzyme and carrier, and conjugation with the carrier) immobilization methods have been used for the attachment of enzymes.^6,8^ Among other methods, physical immobilization via electrostatic interactions is applicable to a large set of enzymes without the need for expensive case-to-case modifications.^9−11^ The method is simple and rapid and allows the immobilization of enzymes at varying pH values and on different supports.^12^

Until now, three types of scaffolds have been explored in search of the optimal host selection for enzyme immobilization: inorganic (e.g., silica),^13,14^ synthetic (e.g., polymethacrylate,^15^ polystyrene,^4^ etc.), and natural polymers.^6,16^ Among them, the natural polymer cellulose and its derivatives stand out as sustainable carriers due to their abundance, biocompatibility, flexibility, biodegradability, pH-dependent solubility, and suitability for chemical modification.^17−21^ Unmodified (native) cellulose in the form of microparticles, microfibers, nanofibers (electrospun membrane), hydrogel, macroporous beads, cotton gauge bandages and fabric, pads, and sponges have been used for various types of enzymes immobilization.^19,22^ Nevertheless, the unmodified scaffolds have limited use for the above-mentioned purpose due to its low reactivity or lack of suitable functional groups.^23,24^ To overcome this, researchers have also used different forms of chemically modified cellulose scaffolds (crystals, beads, films, and fibers) with acetate, sulfate, aldehyde,^24,25^ amino, or carboxylic acid^4^ groups, followed by simultaneous incorporation of either titanium-dioxide,^26^ iron,^27^ or zirconium oxide^28^ nanoparticles. Roberts et al. have also demonstrated the immobilization of enzyme to porous cellulose monoliths via a carbohydrate binding module fusion construct.^29,30^ Despite these numerous types of cellulose-based scaffolds used for enzyme immobilization, they still have certain limitations. For examples, scaffolds made from either conventional cellulose microfibers, cellulose yarns, or pads have low surface-to-mass ratio. On the other hand, scaffolds based on cellulose beads or spheres exhibit a large curvature radius. These characteristics of enzyme carriers may result in low materials attachment, or the immobilized enzyme often cannot effectively contact the substrate.^31^ Scaffolds with specific requirements, such as multiscale porosity, interconnected pores, micro/nanostructures, morphology, charges, mechanical stability, fluid uptake property, and in particular simplified immobilization steps and involvement of no toxic solvents, are therefore necessary in the design of a better immobilization system.

Recently, 3D printing techniques have gained a huge interest in enzyme immobilization as they can rapidly produce self-standing scaffolds with a high degree of complexity and precision. They can also be used to fabricate scaffolds with tailored properties and well-defined architectures that are controlled and consistent in their internal architecture, outer geometry, strand size, and pore size and distribution. By controlling the latter, the dimensional and mechanical properties of the scaffolds can be improved.^21,32,33^ Moreover, 3D printing allows us to fabricate scaffolds from plethora of materials without a material lost and at low cost.^1,34^ However, the research on the development of stable and porous scaffolds through 3D printing from NFC-based composites for enzyme immobilization is still in its infancy. This motivated us to develop chemically cross-linked NFC-based scaffolds from an ink consisting of NFC, CMC, and citric acid and apply them for the single and coimmobilization of enzymes and compare their efficiency. The scaffolds were prepared by the combination of direct-ink-writing (DIW) 3D printing, freeze-drying, and dehydrothermal (DHT) treatment.^32,33^ The latter treatment in the dry state induces a cross-linking of NFC and CMC with CA via an ester bond.^35,36^ The CA cross-linked scaffolds can provide not only an exceptional mechanical and dimensional stability in the aqueous environment but also an adequate porosity or porous structure, hydrophilicity, and fluid uptake capacity. NFC in combination with other charged polysaccharides like CMC not only offers a better environment and compatibility but also exhibits a high surface-to-volume ratio, size (fibril width: 5–20 nm),^32^ specific strength and stiffness, and hydrophilicity. CMC, a derivative of cellulose, is negatively charged and structurally similar to NFC; the latter can thus improve interfacial bonding with NFC and add flexibility to scaffolds^37,38^ Especially, the negative charges of CMC in the scaffold add additional advantages that can favor enzyme immobilization via electrostatic interactions and improved catalytic efficiency.^4,21,39,40^

**Figure 1:**
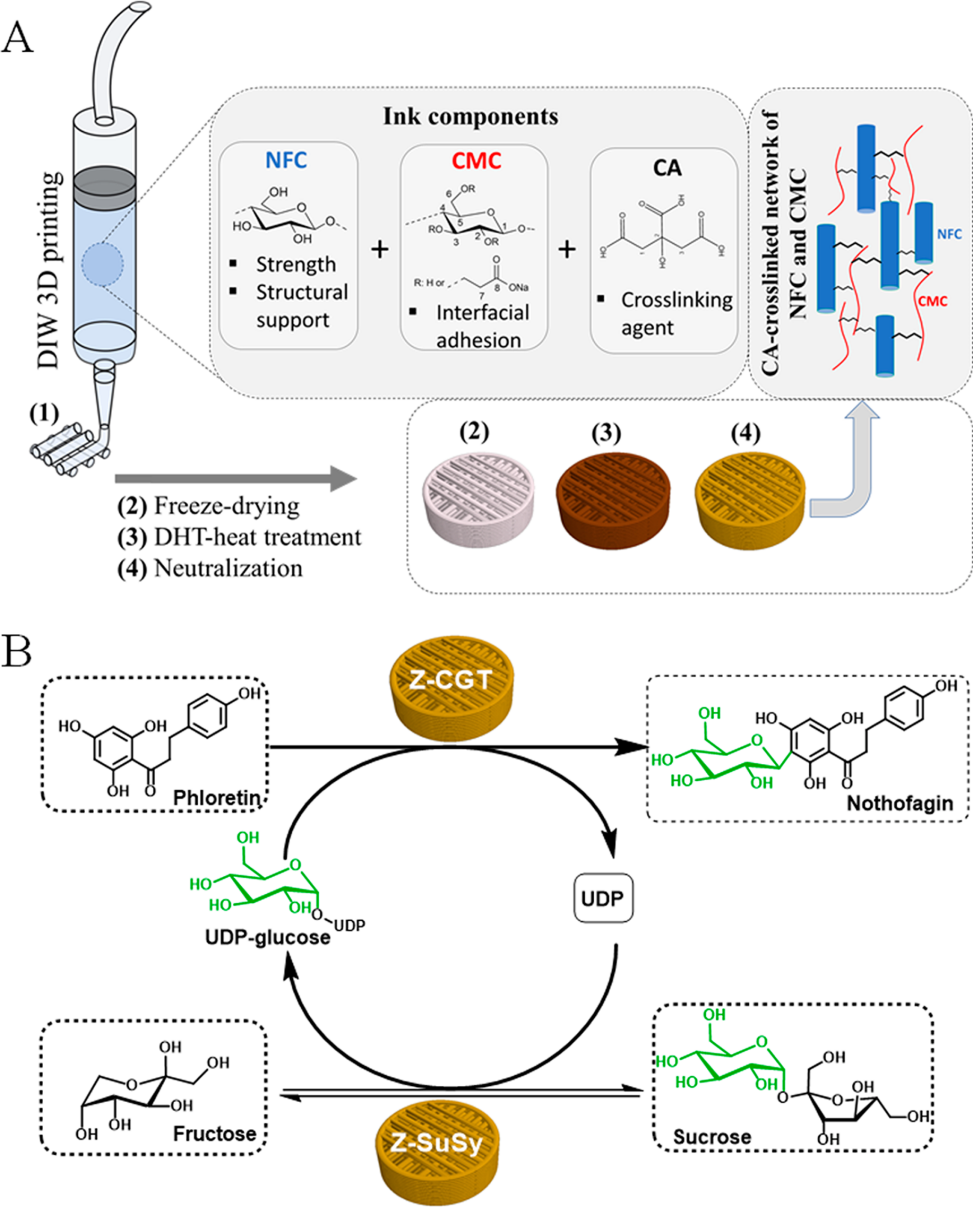
Synthesis of nothofagin using glycosyltransferases immobilized on CA cross-linked NFC/CMC 3D scaffolds. (A) Fabrication of CA cross-linked NFC/CMC porous scaffolds by DIW 3D printing, freeze-drying, DHT treatment, and neutralization. (B) Synthesis of nothofagin via 3′-β-C-d-glucosylation of phloretin using glycosyltransferases (cross-linked NFC/CMC scaffold) from UDP-glucose, synthesized in situ from sucrose and UDP.

Z_basic2_ is a protein module designed to direct adsorption of fusion proteins to a negatively charged surface.^41,42^ This is an engineered variant of the Z-domain of the staphylococcal protein A, originally developed for protein purification. Z_basic2_ is strongly positively charged. Due to multiple arginine residues clustered on one of its sides, the Z_basic2_ exhibits highly localized, positive charge density.^41,42^ These properties enable strong protein binding on anionic supports for enzyme immobilization. A number of studies have shown that enzyme fusions with Z_basic2_ can be immobilized with excellent efficiency, partly due to the fact that the immobilization is orientationally controlled; that is, surface tethering occurs mainly via the Z_basic2_ module.^10,43,44^ The current study was performed with the idea that mechanically and dimensionally stronger NFC/CMC-based scaffolds differing in pore sizes, interconnected morphology or structure, and negative surface charge might present a new class of solid supports for the immobilization of Z_basic2_ enzymes. The enzymes used in this proof-of-principle analysis were from the class of sugar-nucleotide dependent (Leloir) glycosyltransferases. In particular, a *C*-glycosyltransferase (CGT) from rice (*Oryza sativa*) was applied.^10,45^ The enzyme catalyzes the selective 3′-*C*-β-glycosylation of the phloretin (a dihydrochalcone natural product widely distributed in plants; Figure 1B) from uridine 5′-diphosphate (UDP)-glucose. The *C*-glycoside product (nothofagin) is a strong antioxidant and it is prominently found in the rooibos plant as well as in the herbal tea made from it.^46,47^ The second glycosyltransferase studied was soybean (*Glycine max*) sucrose synthase (SuSy).^45,48^ The enzyme catalyzes the conversion of sucrose and UDP to fructose and UDP-glucose (Figure 1B). The reaction is freely reversible at neutral pH.^49^ The SuSy reaction has important uses in glycosyltransferase synthesis to provide UDP-glucose in situ.^48^ The coupled reaction of CGT and SuSy (Figure 1B) has been exploited to produce nothofagin from phloretin and sucrose in the presence of catalytic amounts of UDP.^45,50^ The Z_basic2_ fusions of the two enzymes, here referred to as Z-CGT and Z-SuSy, were reported and their immobilization on synthetic polymer-based supports was shown.^10,11^

Here, we present a systematic characterization of 3D printed porous NFC/CMC scaffolds with focus on application in enzyme immobilization and biocatalysis application. The scaffolds were obtained by DIW 3D printing of NFC/CMC/CA followed by freeze-drying and DHT treatment. The NFC and CMC in the scaffolds were chemically cross-linked with different amounts of CA by DHT treatment at elevated temperature. The morphology, charges, structure, swelling degradation, and mechanical properties of NFC/CMC scaffolds before and after cross-linking with CA were analyzed in detail using various analytical tools. The applicability of the CA cross-linked scaffolds was shown by immobilizing Z-CGT and Z-SuSy individually or by coimmobilizing the two enzymes on the same support. Effects of the support characteristics on immobilization parameters reveal useful binding affinity and immobilized enzyme efficacy for both glycosyltransferases. Activity of the coimmobilized enzyme preparation in the coupled reaction for nothofagin synthesis was also demonstrated. These findings can pave the way to the development and broader application of (nano)polysaccharide-based scaffolds as tunable “designer carriers” for controlled, module-targeted enzyme immobilization. A platform of such supports facilitates selection for optimum host–guest compatibility to achieve the desired performances in biocatalysis applications.

## 2 Experimental Section

### 2.1 Materials

Carboxymethyl cellulose (CMC, DS_COOH_: 0.9; M.wt: 700 kDa), citric acid (CA ≥ 99.5%), and phosphate buffered saline (PBS, pH 7.4) were purchased from Sigma-Aldrich, Austria. Nanofibrillated cellulose (NFC, 3 wt %) was obtained from the University of Maine, USA. Phloretin (>98%), nothofagin (>98%), UDP (97%), HEPES buffer solution, and UDP-glucose (>98%) were from Carbosynth (Berkshire, UK). Ultrapure water (Milli-Q system, Millipore, USA; R > 18.18 M Ω cm) was used for the preparation of all samples.

### 2.2 Ink Preparation

Four types of inks (see Table 1) were prepared from the combinations of NFC, CMC, and CA according to the published protocol.^33^ Briefly, CA at four different concentrations (2.5, 5, 10 g) was mixed with 6 g of CMC and 50 g of NFC (3 wt %) at room temperature. The mixture was stirred continuously for ∼40 min with a mechanical stirrer. An ink of NFC/CMC, but without the addition of CA, was prepared in the same manner as described above. All inks were stored in a refrigerator at 2–8 °C until further use.

**Table 1:** Ink Preparation Based on NFC, CMC, and CA and Their Final Compositions

				final solid content of each component in the inks		
inks	NFC (g)	CMC (g)	CA (g)	NFC (g)	CMC (g)	CA (g)
NC/CA0	50	6	0	1.5	6	0
NC/CA2.5	50	6	2.5	1.5	6	2.5
NC/CA5	50	6	5	1.5	6	5
NC/CA10	50	6	10	1.5	6	10

### 2.3 DIW 3D Printing, Cross-Linking, and Neutralization

All inks mentioned in Table 1 were 3D printed using a BioScaffolder 3.1 (GeSim, Germany). The scaffolds were printed in a circular shape (radius: 0.25 mm; height: 3 mm, number of corners: 100), layer-by-layer fashion. All scaffolds were printed with an inner nozzle diameter of 250 μm and a pressure of 200 kPa. A complete detail of the printing parameters can be found elsewhere.^32,33,51^ For the tensile tests, the scaffolds were printed in the form of a sheet (height: 4 mm and radius: 2.5 cm). Subsequently, all printed scaffolds were freeze-dried and used for cross-linking and neutralization (see below).

All freeze-dried scaffolds were cross-linked by DHT heat treatment at 120 °C for 24 h, as reported elsewhere.^33,52^ The CA cross-linked scaffolds were neutralized with 0.1 M NaOH (60 min), rinsed with Milli-Q water (24 h), and air-dried at room temperature. These dried scaffolds were designated as “NC/CAx” where x is the concentration of CA in wt %.

### 2.4 Enzymes

N-terminal fusions of *Os*CGT (GenBank: FM179712) and *Gm*SuSy (GenBank: AF030231) with the binding module Z_basic2_ are described in Liu et al.^11^ The enzymes referred to as Z-CGT (M.wt: 57.8 kDa, specific activity: 1.38 U/mg) and Z-SuSy (M.wt: 100.7 kDa, specific activity: 1.08 U/mg) were expressed in *Escherichia coli* and purified by reported methods,^9^ and appeared to be ≥95% pure (see Figure S1). The prepared enzyme stock solutions (10–20 mg/mL, in 50 mM HEPES buffer containing 250 mM NaCl, pH 7.5) were frozen (−20 °C) until further use. The enzyme concentration was determined using ROTI9 Quant assay (Carl Roth, reference: BSA) and were stable for 10–20 days (≤5% activity loss). The enzyme solution was thawed no more than three times.

#### 2.4.1 Enzyme Activity Assay

Assays of Z-CGT, Z-SuSy, and coupled Z-CGT-Z-SuSy activity were performed as described in Liu et al.^11,51^ Briefly, the Z-CGT reaction (containing 1.0 mM phloretin and 2.0 mM UDP-glucose), the Z-SuSy reaction (containing 500 mM sucrose and 2.0 mM UDP), and the coupled reaction (containing 1.0 mM phloretin, 500 mM sucrose, and 1 mM UDP) was performed at 30 °C. The reactions were stopped by mixing the sample with ice-cold acetonitrile and allowing them to stand for 15 min. Afterward, the samples were analyzed by HPLC. One unit (U) of enzyme activity corresponds to 1 μmol/min of product (i.e., nothofagin, Z-CGT, and coupled enzyme reactions; UDP-glucose, Z-SuSy reaction).

#### 2.4.2 Enzyme Immobilization

The scaffolds were washed with Milli-Q water and equilibrated in 50 mM HEPES buffer. Afterward, the scaffolds were incubated with ∼300 μL of individual enzyme (∼5 mg protein/mL) for 6 h at 4 °C followed by agitation at 1000 rpm using the Thermo-Mixer C. For enzyme coimmobilization, the mixture of Z-CGT and Z-SuSy (1:3 ratio).^10,11^ The scaffolds were incubated with 600 μL of enzyme mixture (5 mg total protein/mL) for 4 h in the same way as above. Following this, the supernatant was removed and the enzyme immobilized scaffolds were rinsed extensively with 50 mM HEPES buffer. The enzyme activities were measured using the activity assays mentioned in section 2.4.1.

The enzyme activity (protein) was expressed as U/g (mg/g), and the immobilization yield (*Y*_P_,%) and activity (*Y*_A_,%) were determined from the protein (eq 1) and activity (eq 2).
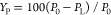
1
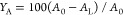
2Where *P*_0_ describes the initial protein concentration (mg/mL), *P*_L_ the remaining protein concentration in the supernatant, and *A*_0_ and *A*_L_ are the corresponding volumetric activities.

The observable activity (expressed as specific activity in U/g scaffold) of the immobilized enzyme (*a*_I_) was determined as described above. Whereas, the specific activity (U/mg) was calculated as the ratio of a known volumetric activity (U/mL, bound active enzyme to the scaffold) and immobilized protein concentration (mg/mL). The effectiveness factor (η) was calculated as the observable and theoretical activity of the immobilized enzyme (eq 3). The effectiveness factor (η_corr_), i.e., the corrected fraction of activity lost in solution during enzyme immobilization is shown in eq 4.
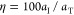
3
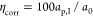
4where *a*_T_ was calculated as (*A*_0_ – *A*_L_)/g scaffold, *a*_0_ (i.e., *A*_0_/*P*_0_) describes the enzyme specific activity in solution before the immobilization, *p*_I_ the immobilized protein (calculated as *P*_0_ – *P*_L_)/g scaffold, and *a*_*p*__,I_ is the ratio *a*_I_ and *p*_I_ (specific activity of the immobilized enzyme). More details of the enzyme immobilization, immobilization parameter and activity can be found in our previous works.^10,11,51^

### 2.5 Analytical Methods

The SEM images of the scaffolds (without sputtering) was acquired using field emission scanning electron microscopy (FE-SEM, Carl Zeiss FE-SEM JSM IT800 SHL) at room temperature and pore sizes were quantified by analyzing the SEM images using ImageJ1.47 software.^53^ Potentiometric charge titration (between 2 < pH < 1) was performed by titrating the sample (1.5 mg mL^–1^) with an automated T70 two-buret titrator (Mettler Toledo, USA) as reported elsewhere.^54^ The ATR-IR spectra (range: 4000–650 cm^–1^), powder X-ray diffraction (scattering angle: 5–70° and a scan rate of 0.02° 2θ s^–1^), and static water contact angle (SWCA, using ultrapure water) measurements of the samples were performed as mentioned in our previous work.^37,55,56^ The swelling kinetics and weight loss of the scaffolds in PBS buffer solution were investigated using a gravimetric method.^32,57^ To determine the surface mechanical properties of dry and wet scaffolds, we performed nanoindentation using a ruby red spherical indenter of 500 μm radius with a Bioindenter (UNHT^3^ Bio). Prior to wet measurements, samples were stored in PBS buffer at 37 °C for 14 days. The details of the procedure can be found elsewhere.^32^ The tensile strength at maximum (kPa) and Young’s modulus (kPa) of the scaffolds were determined using a Shimadzu AGS-X electromechanical universal testing machine as reported in the work of Amornkitbamrung et al.^58,59^ The Z-CGT and Z-SuSy coupled enzyme reaction was analyzed using a Shimadzu model UFLC HPLC as described in our previous work.^51^ Phloretin and its glycoside nothofagin were detected at 288 nm (Figure S2). UDP and UDP-glucose were detected at 262 nm (Figure S2).

## 3 Results and Discussion

### 3.1 Scaffold Preparation and Glycosyltransferases Immobilization

In this work, we aimed to fabricate 3D printed polysaccharide-based scaffolds as a model carrier system. The carriers should provide negative charges, pores, interconnected porous morphology, channels, and adequate dimensional and mechanical stability in an aqueous environment, which are suitable for the immobilization of Z_basic2_ fusions of enzymes, here represented by Z-CGT and Z-SuSy. To this end, we prepared 3D scaffolds from an ink containing a combination of polysaccharides, such as NFC (diameter: ∼20 nm, length: 50–140 nm),^32^ negatively charged CMC,^37^ and the green cross-linker CA. The inks were subjected to DIW 3D printing followed by freeze-drying and DHT treatment (Figure 1A) to obtain free-standing porous and stable scaffolds. DHT treatment performed at an elevated temperature (120 °C) in the dry state (without any solvent), leads to cross-linking, i.e., the formation of ester bonds between the carboxyl groups of CA and the hydroxyl groups of NFC and/or CMC. The concentration of cross-linker CA was varied from 0 to 10 wt % to control or improve the scaffolds properties, such as charges, morphology, structure, pores, swelling, and degradation, in addition to mechanical properties. All CA cross-linked scaffolds were treated with 0.1 M NaOH for 60 min to neutralize the free-acids in the scaffolds. The successfully cross-linked and neutralized scaffolds (acid-free) were further used for the characterization and single-step immobilization (individual/coimmobilization) of Z-CGT and Z-SuSy.

### 3.2 Morphology and Porosity

**Figure 2:**
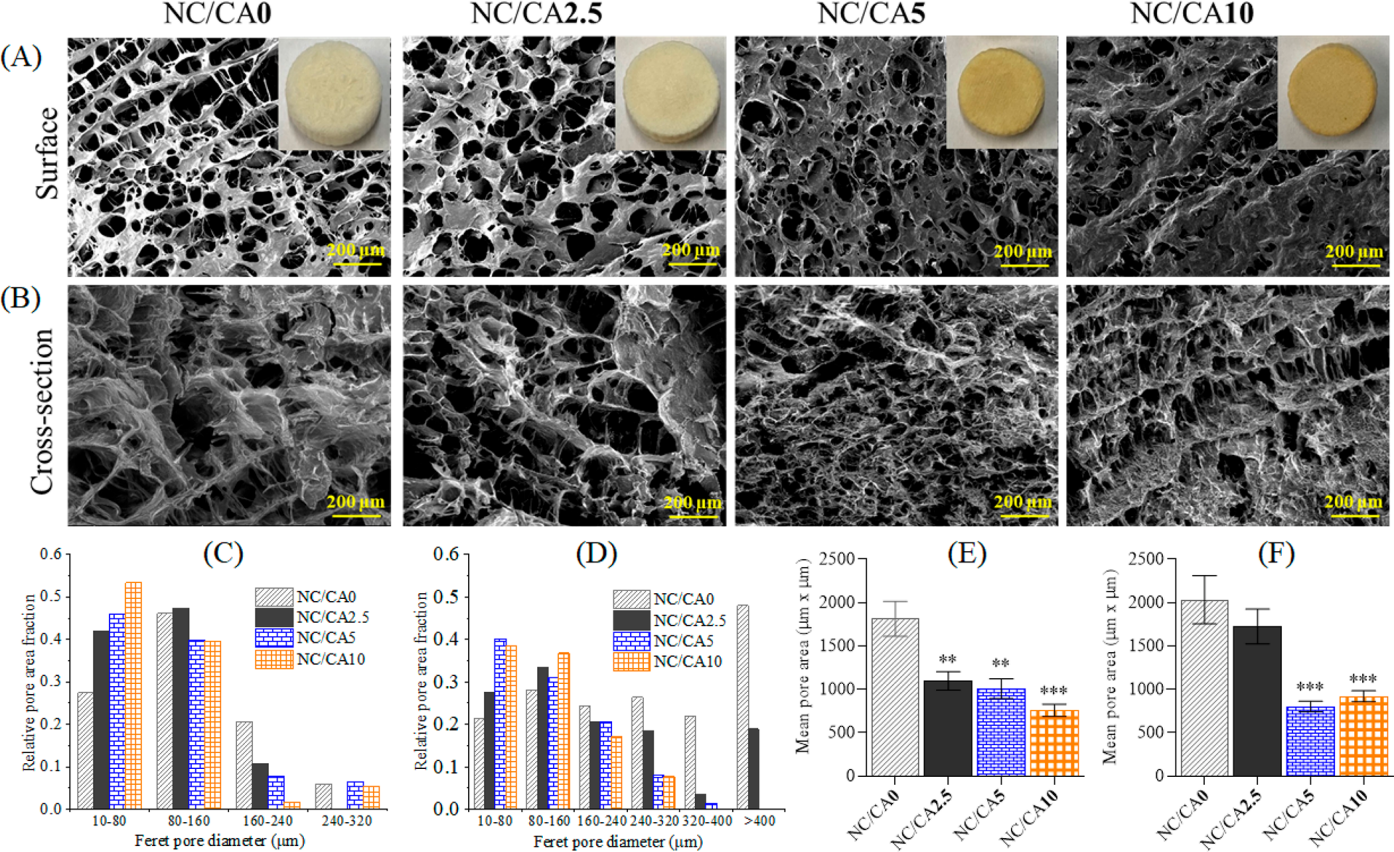
SEM images and pore size analysis of NFC/CMC scaffolds cross-linked with different concentrations of CA (0–10 wt %). (A) Surface and (B) cross-section of dry scaffolds cross-linked without (NC/CA0) and with different concentrations of citric acid (2.5–10 wt %). Magnification in images is 100×. Feret pore diameter (μm) (C) surface and (D) cross-section and mean pore size area (μm × μm) (E) surface and (F) cross-section of non-cross-linked and CA cross-linked scaffolds. Data analysis was done by one-ANOVA with the Dunnett test, values are presented as ± SD; **p < 0.01, ***p < 0.001 (compared to control NC/CA**0**).

The SEM surface and cross-sectional view of the NFC/CMC scaffolds before and after cross-linking with CA are shown in Figure 2. The differences in morphology and pore size between non-cross-linked and cross-linked samples can be clearly seen. The non-cross-linked NC/CA0 scaffold showed a channel-like porous morphology and interconnected macro- and micropores on the surface and in the cross-section compared to CA cross-linked ones. The observed pore size ranged from 10 to 800 μm for both non-cross-linked and cross-linked scaffolds (C and D). The pores at the cross-section were generally larger than those in the surface of the scaffold, except for the NC/CA5 and NC/CA10. For the cross-linked samples, in addition to open to closed morphology, the average pore size or mean pore area (E and F) significantly decreased when the concentration of CA increased. This effect was more pronounced on the scaffold surfaces. It has been suggested that carriers with a pore size of >50 nm may be advantageous to ensure no spatial constraints and effective enzyme immobilization.^60^ Thus, it could be said that the cross-linked scaffolds, which exhibited microporous and macroporous morphology and interconnected structures, are suitable for efficient transport of the necessary components through the scaffolds during the multistep enzymatic cascade reaction.

### 3.3 Structure, Composition, and Charge

**Figure 3:**
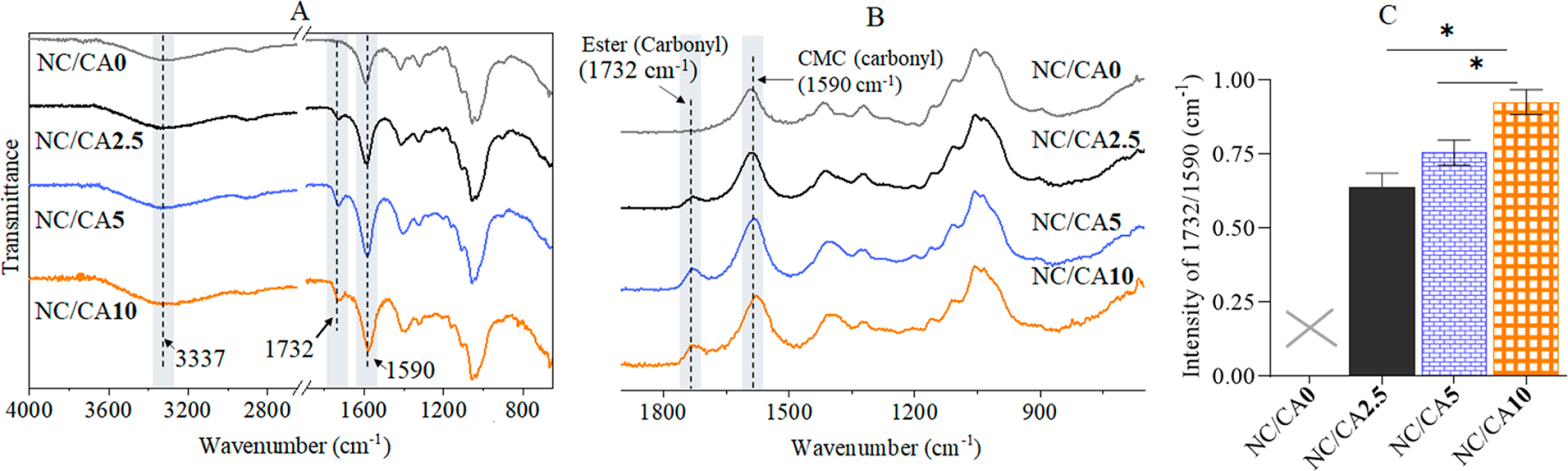
ATR-IR spectra (A) 4000–650 cm^–1^ and (B) 1900–650 cm^–1^ and absorption peak intensity ratio (C) 1732 cm^–1^/1590 cm^–1^ of non-cross-linked and CA cross-linked scaffolds. Data analysis was done by Student’s *t* test with Dunnett test, values are presented as ± SD; *p < 0.05.

Figure 3 shows the ATR-IR spectra of non-cross-linked and CA cross-linked scaffolds. The spectra of the neat polymers (see Figure S3, Supporting Information) show all characteristic peaks for NFC (OH, 3337 cm^–1^; CH, 2910 cm^–1^; C–O, 1100 cm^–1^) and CMC (OH, 3298 cm^–1^; CH, 2910 cm^–1^; COOH, 1590 cm^–1^; C–O, 1100 cm^–1^).^32,37,61^ All these peaks were also observed for the non-cross-linked scaffold (NC/CA0,Figure 3A). Interestingly, in addition to the CMC-carboxyl carbonyl peak at 1590 cm^–1^, a new peak at 1730 cm^–1^ associated with ester carbonyl was observed in the CA cross-linked samples (Figure 3A and B).^57^ This indicates that the carboxyl groups of CA were conjugated to the hydroxyl groups of NFC or CMC via ester bond. We also evaluated the ester cross-linkages semiquantitatively by measuring the absorption intensity ratio (1732 cm^–1^/1590 cm^–1^, see Figure 3C) of the ester carbonyl and carboxyl carbonyl peaks. It could be seen that the intensity of carbonyl ester peak significantly increased with increasing concentration of CA in the scaffold. For scaffolds containing a higher concentration of CA (NC/CA10 and NC/CA5), the intensity of the peak was almost twice as high, indicating that the higher the concentration of CA in the scaffold, the more ester bonds formed between CA and NFC/CMC.

**Figure 4:**
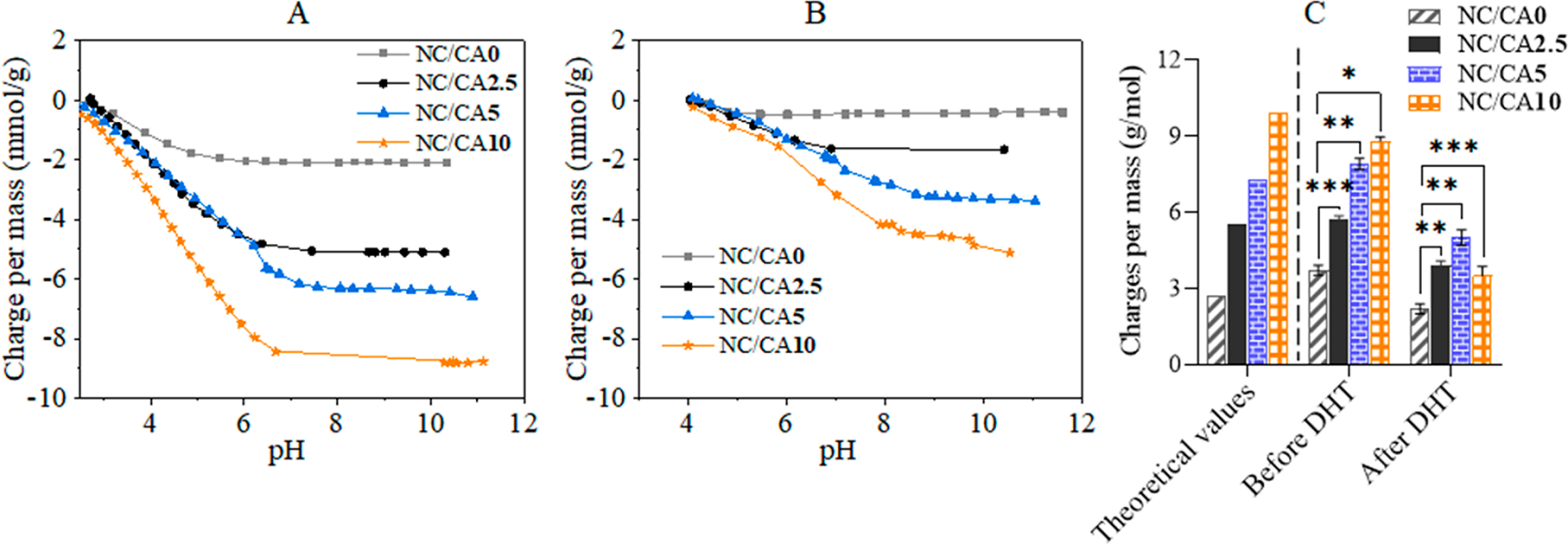
Potentiometric charge titration results for non-cross-linked and CA cross-linked NFC/CMC scaffolds. (A) Freeze-dried and before DHT heat treatment, (B) after DHT heat treatment and neutralization, and (C) comparison of total theoretical and experimentally determined carboxylate charges per mass from panels A and B. Data analysis was done by one-ANOVA with the Dunnett test, values are presented as ± SD; *p < 0.05, **p < 0.01, ***p < 0.001 (compared to control NC/CA**0**).

We also analyzed the (carboxyl) charges of neat polymers and the scaffolds by pH-potentiometric charge titrations and the results are shown in Figure 4. All NFC/CMC curves in Figure 4 exhibit one slope change corresponding to the simultaneous deprotonation of the carboxyl groups of NFC and CMC (pH 2–7 in Figure 4A and pH 2–9 in Figure 4B). This is consistent with the data in the literature.^33,62^ The carboxylate charges of neat NFC and CMC were 0.35 ± 0.05 mmol/g and 3.51 ± 0.17 mmol/g, respectively. A significant difference in the charges between the non-cross-linked and the CA cross-linked scaffolds can be clearly seen. As expected, the total amount of carboxyl groups determined at the point of complete deprotonation (pH > 7) increased from 3.72 ± 0.2 mmol/g to 8.8 ± 0.18 mmol/g as the concentration of CA (0–10 wt %) in the scaffold increased. These total charges are in good agreement with the theoretical values calculated from the combination of NFC, CMC, and CA (Figure 4C). However, the DHT-treated scaffolds showed a significantly reduced charge, which could be due to the consumption of the carboxyl groups of CA in the formation of ester bonds. This also supports the results of IR, where the formation of ester peak was detected at 1730 cm^–1^. The following trend in the reduction of charges was found: NC/CA10 (56%) > NC/CA2.5 (44%) > NC/CA5 (37%) > NC/CA0 (8%). These observed significant differences in the charges between the CA cross-linked scaffolds explain that the accessibility of the charges in the scaffold NC/CA10 was limited compared to the other two cross-linked scaffolds.

The influence of the cross-linking agent and DHT heat treatment on the crystallinity of neat polymers and the NFC/CMC scaffold (Figure S4) was investigated by powder X-ray diffraction (XRD) analysis. As expected, NFC showed four main characteristic diffraction peaks (14.5° (110), 16.4° (200), 20.1° (020), 34.2° (004)), and a pattern corresponding to Cellulose I crystalline structure, while CMC exhibited a broad diffraction pattern and a peak at 2θ = 20° associated with the amorphous structure.^32^ The non-cross-linked scaffold (NC/CA0) showed the typical diffraction peaks of NFC and the main peak of CMC; the latter was masked by the diffraction peak of NFC. In general, all these peaks were also observed in the CA cross-linked samples. Moreover, the intensity of the characteristic peaks of both polymers neither decreased nor disappeared, an indication that the crystallinity of the polymers is well preserved.

### 3.4 Wettability, Swelling, and Weight Loss

**Figure 5:**
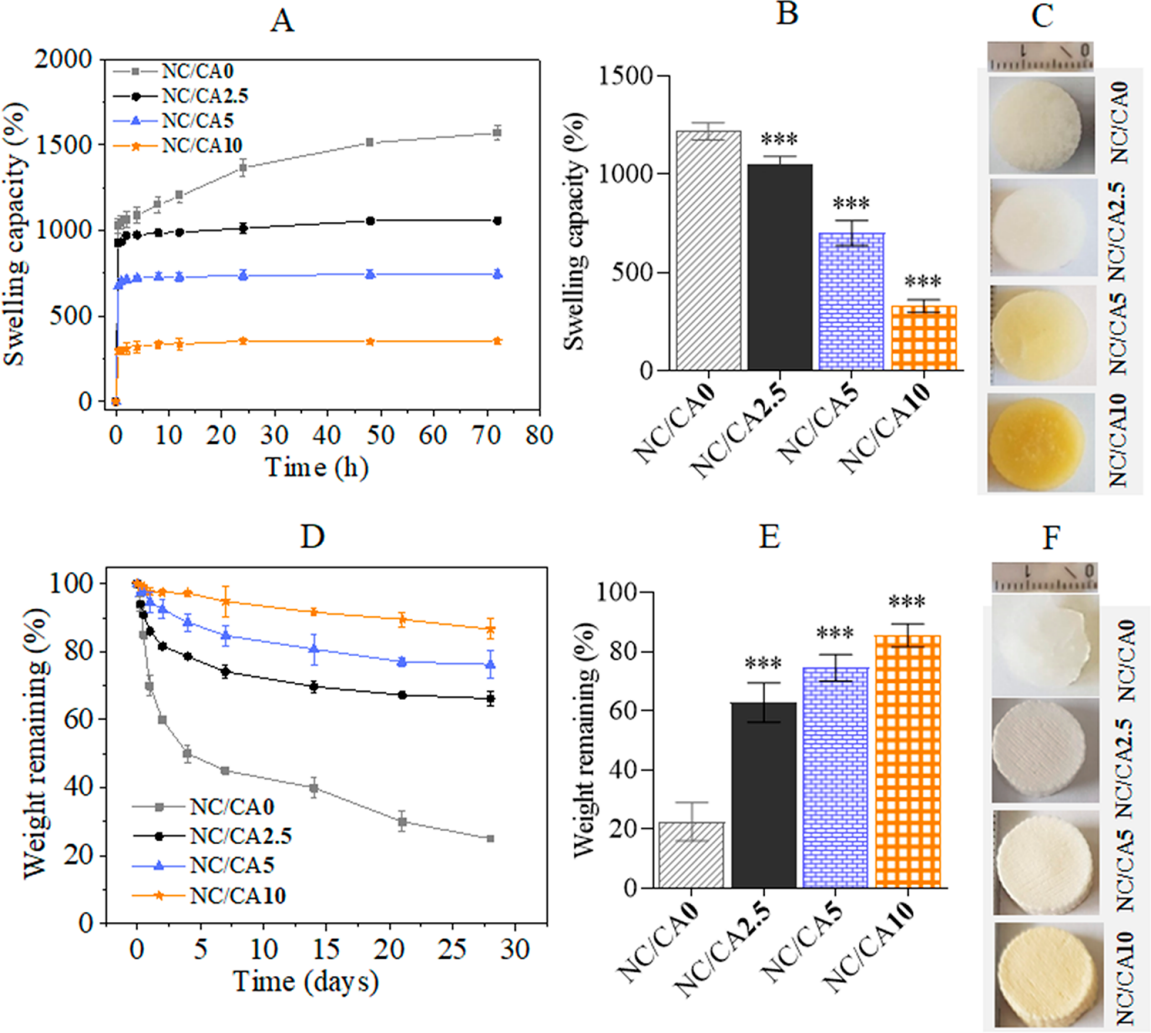
(A, B) Swelling and (D, E) weight loss of NFC/CMC scaffolds cross-linked with different CA concentrations. (C) Images of non-cross-linked and cross-linked NFC/CMC scaffolds taken after 72 h. (F) Images of non-cross-linked and cross-linked NFC/CMC scaffolds taken after the weight loss test at different time points. Data analysis was done by one-ANOVA with the Dunnett test, values are presented as ± SD; ***p < 0.001 (compared to control NC/CA0).

The enzyme immobilization efficiency can be affected by the wettability of the scaffolds. Therefore, we performed static water contact angle (SCA(H_2_O)) measurements for all cross-linked scaffolds. It turned out that it was not possible to determine the (SCA(H_2_O)) values for all samples. The water droplet was absorbed as soon as it touched the surfaces of the scaffold (within a few seconds, see Figure S5). This implies that all scaffolds are very hydrophilic regardless of the cross-linking density achieved with increasing amount of CA. The water absorption or swelling capacity of the scaffold is a crucial factor as it ensures the accessibility of the small molecules to the immobilized enzymes and provides the necessary aqueous environment. Keeping this in mind, we determined the swelling capacity of both non-cross-linked and CA cross-linked scaffolds in PBS under physiological conditions (Temp: 37 °C, pH: 7,4), and the results are shown in Figure 5. The non-cross-linked scaffold showed different swelling kinetics and more fluid uptake than the cross-linked ones. Rapid fluid uptake was observed within 30 min, followed by a steady state almost after 2 and 24 h for cross-linked and non-cross-linked scaffolds, respectively. The swelling capacity of the scaffolds decreased in the following order: NC/CA0: 1570 ± 40 g/g > NC/CA2.5: 1056 ± 17 g/g > NC/CA5: 744 ± 26 g/g > NC/CA10: 355 ± 15 g/g). The observed lower fluid uptake of the CA cross-linked scaffolds indicates that the cross-linking reaction was successful. This correlates with the IR (Figure 3) and SEM (Figure 2) results, where increased ester bond formation and decreased porosity were observed as a function of the CA concentration. It is suggested that the DHT treatment induced a tighter network structure and reduced pore size by cross-linking of hydrophilic groups (e.g., −OH and −COOH) of NFC and CMC, which allowed less water to penetrate into the scaffold structure and thus less fluid uptake.

We analyzed the degradation behavior of the scaffolds in PBS buffer at 37 °C and pH 7.4 (Figure 5D–F), since the shape and stability of the scaffolds in aqueous environments is a critical factor in supporting the long-term activity of the immobilized enzyme. The CA cross-linked scaffold showed a different degradation pattern than the non-cross-linked one (Figure 5D). All scaffolds showed a gradual weight loss for the period of 4 weeks. However, for the non-cross-linked scaffold, a maximum weight loss was observed after 4 weeks, which decreased with the concentration of CA (see Figure 5E). The images of the degraded scaffolds after 4 weeks are shown in Figure 5F. While the non-cross-linked scaffold (NC/CA0) was partially destroyed, no considerable structural or shape change was observed for all CA cross-linked samples. This indicates that cross-linking the functional polymers (NFC and CMC) in the scaffold with CA by DHT treatment improves not only the dimensional stability but also the structural stability of the scaffolds. This kind of scaffolds with versatile properties such as charges, stability, and fluid uptake holds enormous potential to immobilize and retain the specific activities of biological molecules like enzymes.

### 3.5 Mechanical Properties

**Figure 6:**
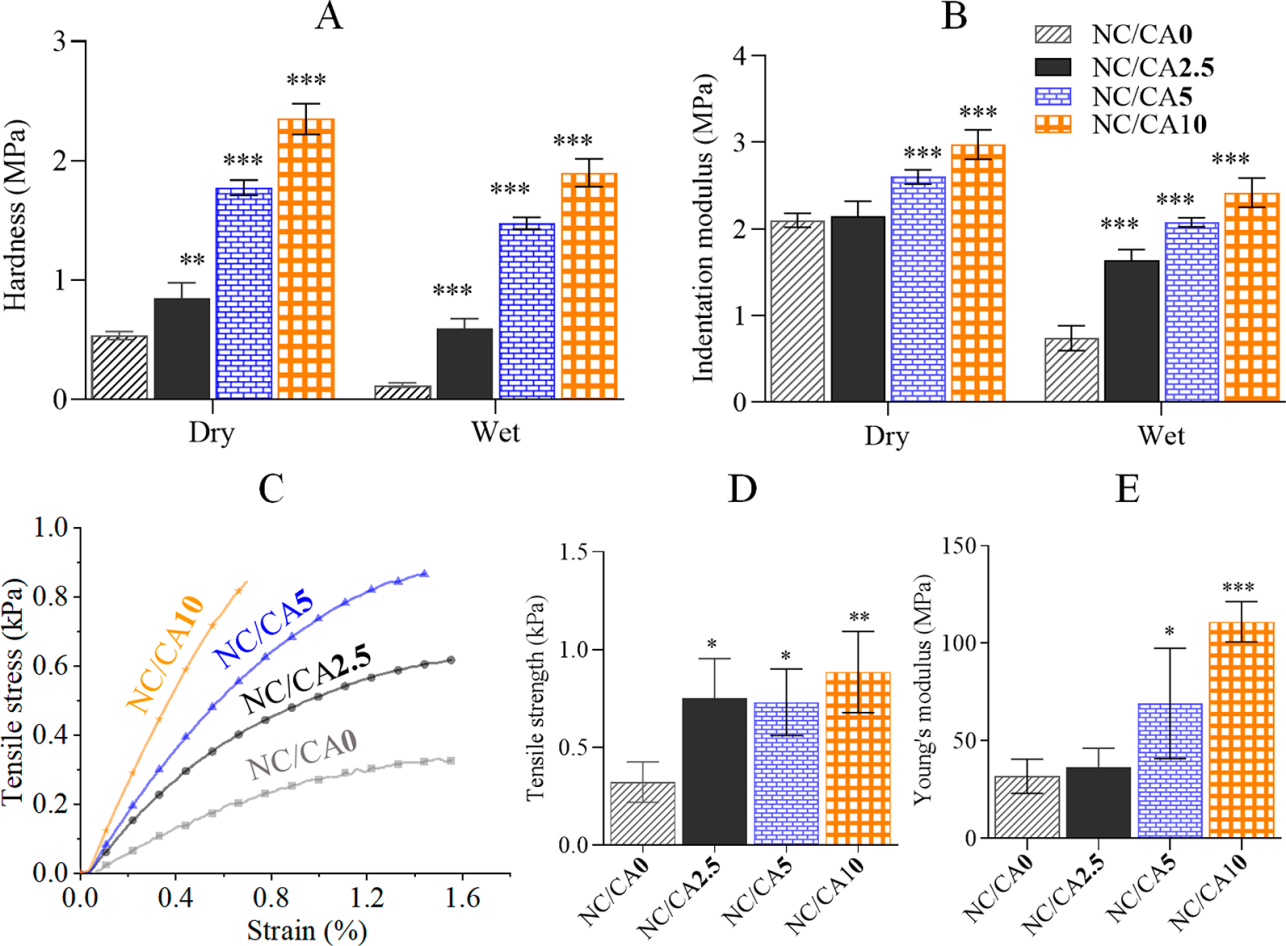
Mechanical properties of non-cross-linked and CA cross-linked scaffolds obtained through nanoindentation ((A) hardness and (B) indentation modulus for dry and wet samples) and tensile testing ((C) stress–strain curves, (D) tensile strength, and (E) Young’s modulus for dry samples) analysis. Data analysis was done by one-ANOVA with the Dunnett test, values are presented as ± SD; *p < 0.05, **p < 0.01, ***p < 0.001 (compared to control NC/CA**0**).

The surface (local) mechanical properties of the scaffolds measured in dry and wet state via indentation experiments are shown in Figure 6. The latter shows a significant difference in hardness and indentation modulus between the cross-linked and the non-cross-linked scaffolds. As expected, both hardness and indentation modulus increased with concentrations of CA (0 to 10 wt %). DHT-assisted CA cross-linking significantly increased the hardness from 538 ± 33 kPa to 2350 ± 129 kPa and the indentation modulus from 2100 ± 81 kPa to 2975 ± 170 kPa. Compared to the dry scaffolds, the mechanical properties of the wet samples (stored in PBS buffer at 37 °C for 2 weeks) were lower as expected and it decreased to 70% for the non-cross-linked (NC/CA0) scaffold. However, the decrease of hardness and indentation modulus was considerably lower for CA cross-linked scaffolds, an indicating the cross-linking with CA stabilized the scaffolds in wet state.

Figure 6C–E shows the stress–strain curves and comparative mechanical properties of the scaffolds measured in dry state. We did not include the results of the scaffolds in the wet state, since false (improper) breaking of the samples was observed during the measurements. It can be seen that the mechanical properties of the scaffolds improved significantly after cross-linking (C). This effect is more obvious as the concentrations of CA increased, being more pronounced at higher CA concentrations (5 and 10 wt %). Increasing the concentrations of CA increased the tensile strength at maximum and the Young’s modulus from 0.3 ± 0.1 to 0.9 ± 0.2 MPa (3-fold) and 31 ± 10 to 111 ± 10 MPa (3.4-fold), respectively. Overall, it can be stated that the CA cross-linked scaffolds with improved mechanical properties (both: surface and bulk) are attractive for extending the recyclability and efficiency of the immobilized enzymes.

## 4 Enzyme Immobilization

### 4.1 Individual Enzyme Immobilization

Purified Z-CGT and Z-SuSy were individually immobilized on the different NFC/CMC scaffolds and assessed for activity (see Figure 1). The NC/CA0 non-cross-linked scaffold was not used due to its low dimensional stability in the aqueous buffer used (see Section 3.4). The protein/dry scaffold mass ratio was constant at 150 mg/g. This corresponded to an activity/unit scaffold mass ratio of 207 U/g (Z-CGT) and 162 U/g (Z-SuSy). A relatively high protein loading was chosen to explore the dynamic binding capacities of the scaffolds used. Each immobilization was characterized by the yields (*Y*) for activity (*Y*_A_) and protein (*Y*_P_) as well as the effectiveness factor of the immobilized enzyme (η) and the results are summarized in Table 2. There was the consistent trend in the results for both enzymes that *Y*_A_ was larger than *Y*_P_. Since purified enzymes were used for immobilization, *Y*_A_ to exceed *Y*_P_ can only be explained by a loss of enzyme activity in solution during the immobilization. This is reflected by a decrease in the specific activity of the enzymes in solution before and after the immobilization (Table S1). The percent loss of specific activity was similar (up to ∼30%) for Z-CGT and Z-SuSy. It did not exhibit a clear dependence on the type of scaffold used (Table S1), even though the NC/CA2.5 scaffold appeared to be more strongly causing activity loss than the NC/CA10 scaffold. Both enzymes were, however, fully stable under the incubation conditions used (1000 rpm agitation rate), in the absence of scaffold. Using Z-CGT, we showed that lowering of the agitation rate to 500 rpm resulted in a clear mitigation of the activity loss in solution (Table S1). The enzyme inactivation is tentatively ascribed to solid–liquid interfacial denaturation of proteins. As the activity parameters (U/g scaffold; U/mg immobilized protein; efficacy) were still superior for immobilization at 1000 rpm (data not shown), we kept the condition of a high agitation rate in all further experiments. The enzyme inactivation in solution implies that *Y*_A_ (obtained according to eq 2) is only apparent. Furthermore, eq 4 must be used to determine the efficacy (η_corr_) of the immobilized enzyme.

**Table 2:** Immobilization of Z-CGT and Z-SuSy on Different CA Cross-Linked NFC/CMC scaffolds

enzyme	carrier	% yield for activity (protein)^a^	observable activity (U/g)^b^	specific activity (U/mg, immobilized protein)^c^	η_corr_ (%)^d^
Z-CGT	NC/CA2.5	41 ± 7(21 ± 3)	14.8 ± 0.7	0.49 ± 0.09	35 ± 6
	NC/CA5	43 ± 5(22 ± 2)	16.5 ± 1.5	0.50 ± 0.08	37 ± 5
	NC/CA10	37 ± 5(21 ± 4)	10.3 ± 0.6	0.36 ± 0.06	26 ± 4
Z-SuSy	NC/CA2.5	48 ± 7(23 ± 3)	6.6 ± 0.3	0.19 ± 0.03	18 ± 3
	NC/CA5	44 ± 6(22 ± 2)	5.7 ± 0.2	0.17 ± 0.01	16 ± 1
	NC/CA10	38 ± 5(21 ± 3)	4.5 ± 0.8	0.14 ± 0.02	13 ± 1

a Calculated using eqs 1 and 2. Note that *Y*_A_ is apparent because it includes the effect of enzyme inactivation in solution during the immobilization.

b *a*_I_ as defined in the Experimental Section.

c *a*_*p*__,I_ as defined in the Experimental Section.

d Calculated using eq 4.

For Z-CGT, the maximum observed activity was ∼16.5 U/g with the NC/CA5 scaffold. The corresponding *Y*_P_ and η were 22% and 37%, respectively. The specific activity of the immobilized Z-CGT was 0.50 U/mg. The NC/CA2.5 scaffold gave comparable results. Although binding (*Y*_P_) was retained by the NC/CA10 scaffold, the activity parameters were decreased (Table 1). For Z-SuSy, the *Y*_p_ (21–23%) was almost similar to that of Z-CGT. The maximum η_corr_ (∼18%) and observable activity with NC/CA2.5 scaffold (∼6.6 U/g carrier) were smaller for Z-SuSy than for Z-CGT. The specific activity of immobilized Z-SuSy was only ∼0.19 U/mg. However, as with Z-CGT, the scaffold cross-linked with the highest CA concentration showed the lowest performance.

**Figure 7:**
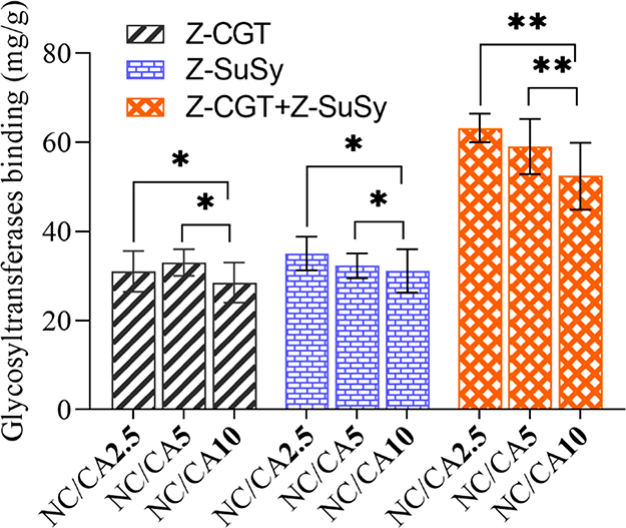
Calculated amount of bound glycosyltranferases on the CA cross-linked NFC/CMC scaffolds after individual and coimmobilzation with Z-CGT and Z-SuSy. Data analysis was done by a Student’s *t* test with Dunnett test, values are presented as ± SD; *p < 0.05 or **p < 0.01.

Figure 7 shows the protein binding on each scaffold. These immobilization results can arguably be corroborated with the evidence from charge titration and SEM. It is suggested that the cross-linked scaffolds (NC/CA2.5 and NC/CA5) with more charges and open pores and interconnected structure favored enzyme adsorption with retention of activity than the NC/CA10 scaffold, which exhibited more closed morphology, fewer pores, and reduced charge. No significant differences in protein binding were observed between scaffolds NC/CA2.5 and NC/CA5. This indicates that a saturation of adsorbed enzyme was reached already for NC/CA**2**.5 or the charges in the scaffold were less accessible for the enzyme as the cross-linking density increased as a function of CA concentration. This can be further noticed for highly cross-linked NC/CA10 scaffold that showed further less protein binding. It is interesting that the structural changes of solid support represented in the scaffolds used are not highly important for overall binding expressed in *Y*_p_, yet they appear to influence the functional efficiency of the immobilization. The enzyme immobilization on the NC/CA scaffolds can be compared to earlier immobilization results on ReliSorb SP400 (a polymethacrylate-based porous support with sulfonate groups).^11^ Preparations of Z-CGT and Z-SuSy immobilized individually to an observable activity (U/g) comparable to ones in Table 2 exhibited an efficacy (η) well over 50%. Intuitive explanation for the difference in η is that Z-enzymes bind in a more defined, activity-retaining orientation via their Z_basic2_ module on ReliSorb SP400 as compared to the cross-linked NC/CA scaffolds. Surface charge analysis rules out the tentative suggestion that NC/CA scaffolds might provide a lower amount/density of negatively charged groups than ReliSorb SP400.^63^ In fact, the specific density of negative charges of the scaffolds is about 10-times higher than it is on ReliSorb SP400. The findings are in accordance with previous evidence on Z-enzyme immobilization done with other enzymes (D-amino acid oxidase;^64^ sucrose phosphorylase),^65^ showing that sulfonate groups are superior to carboxylate groups in promoting enzyme adsorption via the Z_basic2_ module. It is tempting to speculate that sulfonate groups enable different, and more specific, molecular interactions with the arginine residues of Z_basic2_ than carboxylate groups do. While speculative at this time, the incorporation of sulfonate groups might constitute a strategy to enhance the performance of NC/CA scaffolds for Z-enzyme immobilization.

### 4.2 Glycosyltransferase Coimmobilization for Coupled Enzyme Reaction

**Figure 8:**
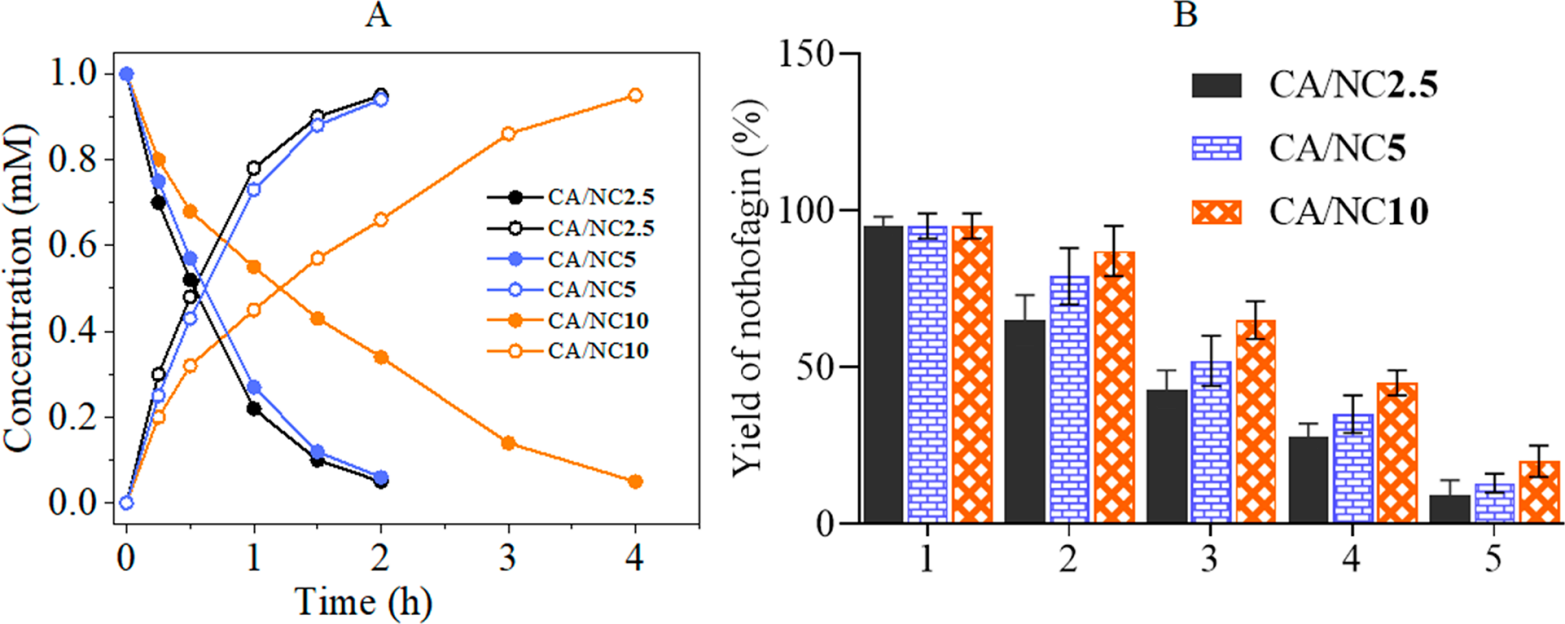
Glycosylation performed with coimmobilized glycosyltransferases. (A) Synthesis of nothofagin by coimmobilized Z-CGT and Z-SuSy on different CA cross-linked NFC/CMC scaffolds (closed circle, phloretin; open circle, nothofagin). (B) Repeated reaction as in panel A with reuse of solid catalyst. Each cycle lasted 2 h (except for NC/CA10 scaffold) and solid catalyst was recovered by centrifugation after each cycle. Standard deviation in B shows *N* ≥ 3.

Glycosyltransferase-catalyzed synthesis is usually done in a coupled enzyme reaction that involves in situ supply of the sugar nucleotide substrate.^48,49^Figure 1B shows the coupled reaction of Z-CGT and Z-SuSy for nothofagin synthesis. In order to perform the coupled reaction with immobilized enzymes, it is often useful to apply coimmobilization (i.e., the immobilization of both enzymes on the same support).^66^ Work done with Z-CGT and Z-SuSy immobilized on ReliSorb SP400 reveals that coimmobilization can provide a 2.5-fold benefit in terms of overall activity.^9^ Z-CGT and Z-SuSy were therefore coimmobilized on the CA cross-linked NFC/CMC scaffolds, offering a 207 U/g carrier and a 162 U/g carrier, respectively. The total protein loading was doubled (300 mg/g carrier) compared to the individual immobilizations (Z-CGT: 150 mg/g; Z-SuSy: 150 mg/g). The *Y*_P_ for the coimmobilization was 20%, almost identical to the *Y*_P_ of the individual immobilizations (Table 2). The result is interesting for it implies that the individual immobilizations have not reached the maximum protein binding capacity of the scaffold. It involves the additional suggestion that Z-CGT and Z-SuSy applied together for coimmobilization do not compete mutually for binding to a limiting amount of adsorption sites on the scaffold. If they did, one would expect the *Y*_P_ of the coimmobilization to be distinctly lower than the individual *Y*_P_. Figure 8 shows that the overall *Y*_P_ for coimmobilization followed the trend to decrease with increasing CA content. The coimmobilized enzyme preparations were used in a coupled reaction to examine whether they can promote nothofagin formation from sucrose, phloretin, and UDP. The conditions used for the proof-of-principle test restricted the reaction to a single glucosyl transfer to phloretin via UDP-glucose. As shown in Figure 8A, the time courses of nothofagin release were almost superimposable or similar activity was determined for both enzyme immobilized NC/CA2.5 and NC/CA5 scaffolds. With the latter two scaffolds, complete conversion of phloretin to nothofagin was achieved within 2 h, whereas it was slower in the case of NC/CA10, consistent with the activity measurements (Table S2).

We also tested the recyclability of the scaffolds coimmobilized with enzymes. The experiment was performed up to 5 consecutive cycles under the same conditions as in Figure 8A. It can be seen that the nothofagin yield gradually decreased with each cycle. This was noticed for all scaffolds but was more pronounced for the scaffold cross-linked with 2.5 and 5% CA. The observed higher yield of nothofagin in each cycle can be related to the higher stability of the both immobilized enzymes. However, it should be mentioned that the observed enzyme activity for our 3D printed scaffolds was still lower than the activity reported for other negatively charged (sulfonated) carriers such as Relisorb SP400 beads coimmobilized with glycosyltransferases.^10,11^ Although we cannot explain the reason for this behavior at the moment, it is obvious that the differences in pore sizes, structure, stability, and accessibility of charges in aqueous environments may play a crucial role in improving enzyme loading and thus nothofagin synthesis. Detailed investigation is currently underway, and discussion at this point would be premature.

## 5 Conclusions

3D printed porous scaffolds as sustainable carriers are required to improve enzyme stability and activity. In this context, we fabricated polysaccharide-based porous scaffolds for one-step immobilization of glycosyltransferases and continuous synthesis of nothofagin in an aqueous environment. The scaffolds, consisting of NFC, CMC, and CA, were fabricated by a combination of DIW 3D printing and freeze-drying techniques. The NFC and CMC polymers in the NFC/CMC scaffolds were cross-linked via ester bonds with CA at different concentrations (0 to 10 wt %) at an elevated temperature (120 °C) in the dry state (without solvent). This was done by DHT heat treatment. It was found that the cross-linking density achieved with increasing CA concentration influenced the morphology, charges, structure, fluid uptake and mechanical properties. This was also reflected in the individual and coimmobilization of glycosyltransferases. Scaffolds cross-linked with a lower CA concentration resulted in higher enzyme loading and activity. Maximum activity i.e., synthesis of nothofagin (*ca*. 15–17 U/g) from phloretin was found for NC/CA2.5 and NC/CA5 immobilized with individual enzyme. In the case of coimmobilization, the observed activity was in the range of 9–13 U/g. All scaffolds coimmobilized with Z-CGT and Z-SuSy showed recyclability up to a maximum of five cycles, but the activity gradually decreased with each cycle. It can be concluded that the NC/CA2.5 and NC/CA5 scaffolds, which showed porous morphology, open and interconnected pores, and high negative charge density, are highly advantageous or more suitable for individual enzyme immobilization. Whereas the highly cross-linked, less charged, and less porous scaffold NC/CA10 is more suitable for coimmobilization and recyclability of the immobilized enzyme. Indeed, the observed activity of the NFC/CMC scaffolds immobilized with Z-CGT and Z-SuSy was not comparable to the results obtained for the same enzymes immobilized on Relisorb SP400 beads. This disadvantage should be remedied by introducing alternative charged groups, like sulfonate. This will be done in our future studies. In general, 3D printed scaffolds prepared with a green approach could have great potential for enzyme biocatalyst preparation via immobilization.

## Supporting Information Available

SDS polyacrylamide gel (with cell extracts and purified Z_basic2_ enzymes), HPLC quantification of phloretin and nothofagin UDP and UDP-glucose, ATR-IR spectra of NFC and CMC, XRD spectra of polymers and NFC/CMC scaffolds, enzyme stability (Z-CGT and Z-SuSy in solution), and coimmobilization of Z-CGT and Z-SuSy on cross-linked scaffolds (PDF)
