# Supplementary material for: 3D Printed Porous Nanocellulose-Based Scaffolds As Carriers for Immobilization of Glycosyltransferases
Source: ACS Appl Bio Mater. 2022 Dec 5;5(12):5728–40. doi: 10.1021/acsabm.2c00763 (PMC9768809; doi:10.1021/acsabm.2c00763)
Supplement: Supplementary file 1 — mt2c00763_si_001.pdf [file mt2c00763_si_001.pdf]

Supporting Information

3D printed porous nanocellulose-based scaffolds as carriers for immobilization of glycosyltransferases

Florian Lackner[†,1,≠], Hui Liu[‡,≠], Andreja Dobaj Štiglic[$], Matej Bračič[$], Rupert Kargl[†,β,1], Bernd Nidetzky[‡,§], , Tamilselvan Mohan[†,$,1*] Karin Stana Kleinschek[†,β,1,*]

[†]*Graz University of Technology, Institute for Chemistry and Technology of Biobased System (IBioSys), Stremayrgasse 9, 8010 Graz, Austria.*

[‡]*Graz University of Technology, Institute of Biotechnology and Biochemical Engineering, NAWI, Petersgasse 12, 8010 Graz, Austria.*

[$]*University of Maribor, Faculty of Mechanical Engineering, Laboratory for Characterization and Processing of Polymers, Smetanova Ulica 17, 2000 Maribor, Slovenia.*

[β]*University of Maribor, Institute of Automation, Faculty of Electrical Engineering and Computer Science, Koroška cesta 46, 2000 Maribor, Slovenia.*

[§]*Austrian Centre of Industrial Biotechnology (ACIB), Graz, Austria.*

[1]*Members of the European Polysaccharide Network of Excellence (EPNOE).*

Corresponding Author

*tamilselvan.mohan@tugraz.at; Phone number: +43 316 873 - 32076

*karin.stanakleinschek@tugraz.at; Phone number: +43 316 873 - 32070

Table S1. Stability of Z-CGT and Z-SuSy in solution during enzyme immobilization on 3D scaffolds.

Enzyme	Carrier	Specific activity in free enzyme (U/mg)	Specific activity in supernatant (U/mg)	Activity loss in supernatant (%)
Z-CGT[a]	NC/CA**2.5**	1.38 ± 0.2	1.05 ± 0.08	24 ± 6
	NC/CA**5**		1.02 ± 0.05	26 ± 4
	NC/CA**10**		1.12 ± 0.06<br>1.27 ± 0.02[c]	19 ± 5<br>8 ± 1[c]
Z-SuSy[b]	NC/CA**2.5**	1.08 ± 0.1	0.74 ± 0.07	31 ± 6
	NC/CA**5**		0.79 ± 0.06	27 ± 5
	NC/CA**10**		0.83 ± 0.02	23 ± 2

[a] Reaction conditions: 1.0 mM phloretin, 2.0 mM UDP-glucose, 50 mM KCl, 13 mM $MgCl_2$, 4% DMSO, 50 mM HEPES buffer (pH 7.5)

[b] Reaction conditions: 500 mM sucrose, 2.0 mM UDP, 50 mM KCl, 13 mM $MgCl_2$; 50 mM BisTris buffer (pH 6.5).

[c] Experiment performed at 500 rpm agitation rate.

The enzyme loading was 207 U/g carrier and 162 U/g carrier for Z-CGT and Z-SuSy, respectively.

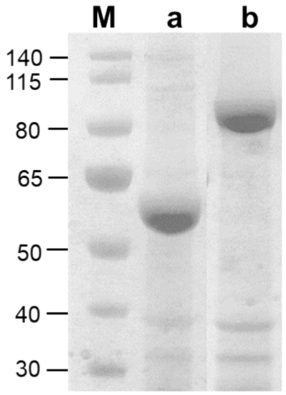

Figure S1. SDS polyacrylamide gel with the cell extracts and purified Z_{basic2} enzymes shown. M, PageRuler™ Prestained Protein Ladder (10-140 kDa); a, purified Z-CGT (57.8 kDa); b, purified Z-SuSy (100.7 kDa).

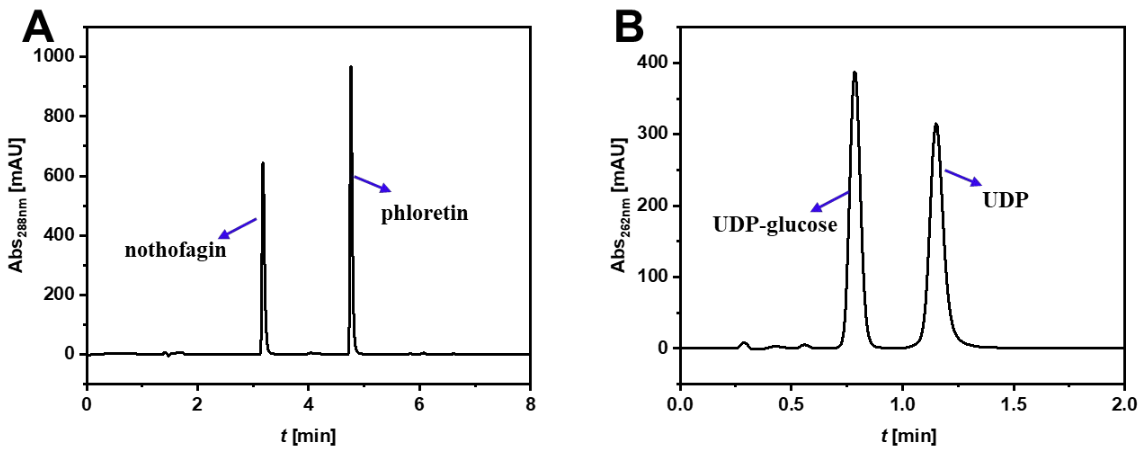

Figure S2. Analysis with HPLC. A: Reversed phase C-18 HPLC with UV-detection at 288 nm for the quantification of phloretin and nothofagin. B: Reversed phase C-18 HPLC with UV-detection at 262 nm using ion-pairing by TBAB is applied for quantification of UDP and UDP-glucose.

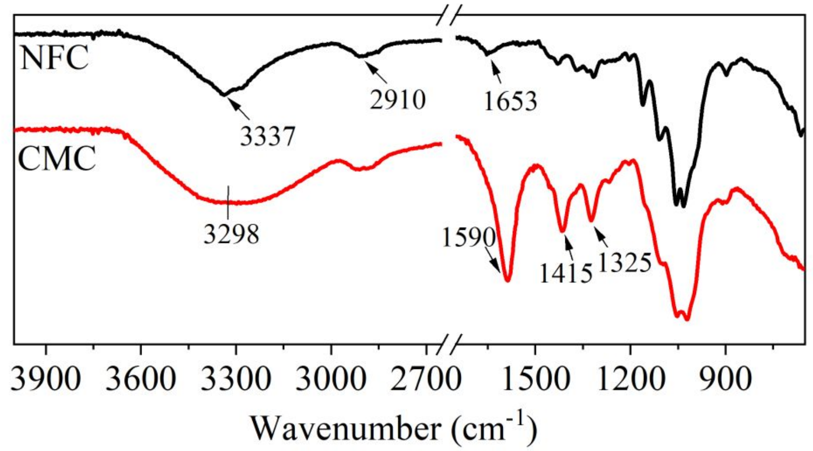

Figure S3. ATR-IR spectra of nanofibrillated cellulose (NFC) and carboxymethyl cellulose (CMC).

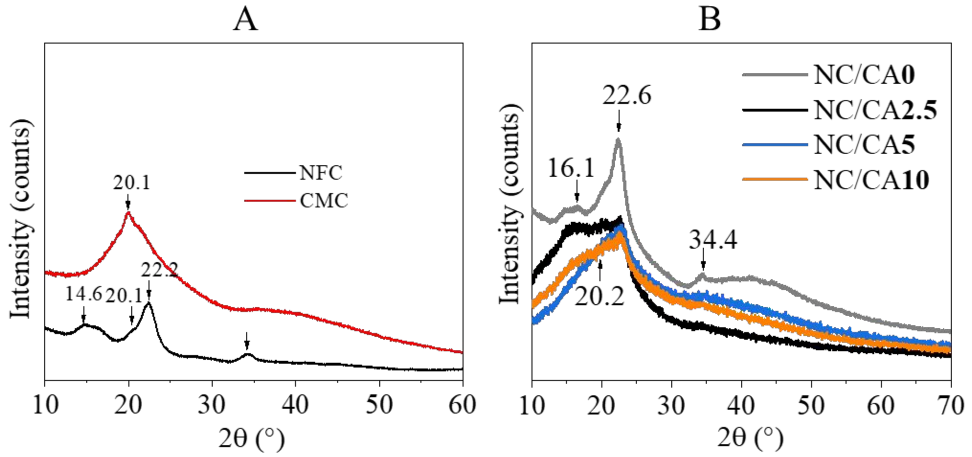

Figure S4. XRD spectra of neat polymers (A: NFC and CMC) and NFC/CMC scaffolds crosslinked with and without citric acid (B).

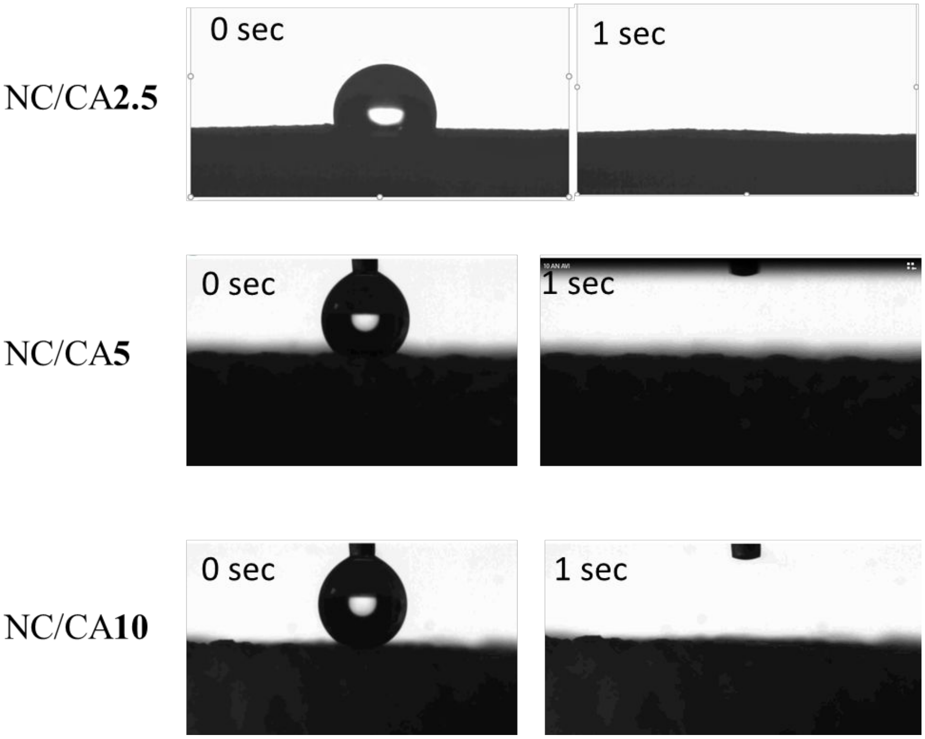

Figure S5. Photo images of water contact angle measurements of crosslinked scaffolds.

Table S2. Co-immobilization[a] of Z-CGT and Z-SuSy on different CA-crosslinked NFC/CMC scaffolds

Carrier	% Yield for activity (protein)	Observable activity (U/g)[c]	Specific activity (U/mg, immobilized protein)	η (%)	Observable activity (U/g)[d]
NC/CA2.5[b]	42 ± 5 (22 ± 2)	13 ± 0.8	0.39 ± 0.01	28 ± 1	5.9 ± 0.7
	46 ± 5 (22 ± 2)	5.1 ± 0.5	0.15 ± 0.01	14 ± 1	
NC/CA5[b]	43 ± 4 (22 ± 2)	14 ± 0.6	0.46 ± 0.01	34 ± 1	5.2 ± 0.2
	43 ± 2 (22 ± 2)	4.4 ± 0.5	0.14 ± 0.01	13 ± 1	
NC/CA10[b]	40 ± 4 (21 ± 1)	9.2 ± 0.2	0.29 ± 0.01	21 ± 1	3.6 ± 0.3
	42 ± 3 (21 ± 1)	3.1 ± 0.3	0.10 ± 0.01	9 ± 1	

[a] Co-immobilization procedure: 10 mg dry 3D scaffold carrier was incubated with freshly co-immobilized enzyme of Z-CGT and Z-SuSy (600 µL, ~5 mg total protein/mL) at 4°C and 1000 rpm for 4 h. The purified Z-CGT and Z-SuSy were mixed in an activity ratio of ~1.3 based on earlier work[1], offering an enzyme loading of 207 U/g carrier and 162 U/g carrier, respectively. The total protein loading used was 300 mg/g carrier (Z-CGT: 1.5 mg; Z-SuSy: 1.5 mg; dry 3D scaffold: 10 mg).

[b]The top row shows the data for Z-CGT, the bottom row that for Z-SuSy.

[c] The observable activity for individual enzyme preparation is shown. Z-CGT: 1.0 mM phloretin, 2.0 mM UDP-glucose, 50 mM KCl, 13 mM $MgCl_2$, 4% DMSO, 50 mM HEPES buffer (pH 7.5); 30°C, 1000 rpm; Z-SuSy: 500 mM sucrose, 2.0 mM UDP, 50 mM KCl, 13 mM $MgCl_2$; 50 mM BisTris buffer (pH 6.5); 30°C, 1000 rpm.

[d] The observable activity for co-immobilized enzyme of Z-CGT and Z-SuSy preparation is shown. Reaction conditions: 1.0 mM phloretin, 500 mM sucrose, 1.0 mM UDP, 50 mM KCl, 13 mM $MgCl_2$, 20% DMSO, 1.3 mg/ml BSA and 50 mM HEPES buffer (pH 7.5).

References

1. Liu, H.; Tegl, G.; Nidetzky, B., Glycosyltransferase co-Immobilization for natural product glycosylation: cascade biosynthesis of the C-glucoside nothofagin with efficient reuse of enzymes. *Advanced Synthesis & Catalysis* **2021,** *363* (8), 2157-2169.
